# Contrasting effects of NADPH oxidases on the fungal hyphae growth and immune responses in *Pleurotus ostreatus*

**DOI:** 10.3389/fmicb.2024.1387643

**Published:** 2024-06-19

**Authors:** Huiping Li, Jiachun Zhu, Zihao Li, Ping Xu, Lin Ma, Yajie Zou, Shaoxuan Qu, Xiaoqin Wu

**Affiliations:** ^1^Co-Innovation Center for Sustainable Forestry in Southern China, College of Forestry, Nanjing Forestry University, Nanjing, Jiangsu, China; ^2^Institute of Vegetable Crops, Jiangsu Key Laboratory for Horticultural Crop Genetic Improvement, Jiangsu Academy of Agricultural Sciences, Nanjing, Jiangsu, China; ^3^Institute of Life Science, Jiangsu University, Zhenjiang, Jiangsu, China; ^4^Institute of Agricultural Resources and Regional Planning, Chinese Academy of Agricultural Sciences, Beijing, China

**Keywords:** NADPH oxidases, oyster mushroom, H_2_O_2_, fungal defense, ROS

## Abstract

*Pleurotus ostreatus* is one of the most consumed mushroom species, as it serves as a high-quality food, favors a rich secondary metabolism, and has remarkable adaptability to the environment and predators. In this study, we investigated the function of two key reactive oxygen species producing enzyme NADPH oxidase (PoNoxA and PoNoxB) in *P. ostreatus* hyphae growth, metabolite production, signaling pathway activation, and immune responses to different stresses. Characterization of the Nox mutants showed that PoNoxB played an important role in the hyphal formation of the multicellular structure, while PoNoxA regulated apical dominance. The ability of *P. ostreatus* to tolerate a series of abiotic stress conditions (e.g., osmotic, oxidative, membrane, and cell-wall stresses) and mechanical damage repair was enhanced with PoNoxA over-expression. PoNoxB had a greater responsibility in regulating the polysaccharide composition of the cell wall and methyl jasmonate and gibberellin GA1 biosynthesis, and improved mushroom resistance against *Tyrophagus putrescentiae*. Moreover, mutants were involved in the jasmonate and GA signaling pathway, and toxic protein defense metabolite production. Our findings shed light on how the oyster mushroom senses stress signals and responds to adverse environments by the complex regulators of Noxs.

## 1 Introduction

Reactive oxygen species (ROS) are important signaling molecules that regulate development, aging, environmental stress responses and host defense in eukaryotic cells and tissues (Aguirre et al., 2005; Davalli et al., 2016). ROS, particularly hydrogen peroxide (H_2_O_2_), were initially considered to be cytotoxic and continuously produced through diverse cellular responses to stimuli (Mittler, 2017). More recently, ROS have been recognized as key signaling molecules that regulate gene expression, hormone production, and secondary metabolite biosynthesis (Rohlfs, 2015; Xia et al., 2015; Tian et al., 2018; Shi et al., 2022). Moreover, ROS are involved in physiological and pathological processes in fungi, including fungal sexual reproduction, mechanical stress responses, biosynthesis of heat stress resistance triterpenes, host infection, and antipredator defenses (Liu et al., 2018; Wang et al., 2022).

NADPH oxidases (Noxs), which mediate the release of ROS production, comprise four conserved heme-binding histidine-transferring electrons from NADPH to oxygen, resulting in free radicals, and non-radical oxidant species, such as the superoxide anion (O${\;}_{2}^{-}$) and hydrogen peroxide (H_2_O_2_). Three Nox isoforms (NoxA, B, and C) have been identified in fungi. NoxA and NoxB (also known as Nox1 and Nox2) are found in a wide range of fungal taxa, while NoxC is not strictly conserved and mostly found in phylum Ascomycota (Wang et al., 2020; Ogboo et al., 2022). Phylogeny indicates fungal calcium-independency as NoxA and NoxB diverge from the oldest classified metazoan calcium independent system in eukaryotes (e.g., NOX4). This allows for a sophisticated regulation through protein-protein interactions in certain cellular pathways to mediate ROS production (Wang et al., 2020). By contrast, NoxC has a longer N-terminal domain with a putative calcium-binding EF-hand motif. This motif is found in both human Nox5 and plant respiratory burst oxidase homologs (Rboh) (Medina-Castellanos et al., 2014, 2018; Ogboo et al., 2022). Filamentous pathogenic fungi successfully and effectively infect host cells, reproduce, and transmit by destroying host innate immunity (Tudzynski et al., 2012). Numerous studies have shown that NoxA and NoxB are involved in appressorium formation, aerial hyphal growth, female sterility, sexual spore germination, and fruiting body differentiation (Tudzynski et al., 2012; Lopez-Moya et al., 2021; Liu et al., 2022). In several pathogenic fungi (e.g., *Curvularia lunata, Magnaporthe oryzae*, and *Colletotrichum gloeosporioides*), NoxA is localized to the plasma membrane and involved in sexual reproduction and host infection by regulating the production and distribution of H_2_O_2_, as well as the mitogen-activated protein kinase (MAPK) and cyclic adenosine monophosphate-protein kinase A (cAMP-PKA) signaling pathways (Lara-Rojas et al., 2011; Adl et al., 2019; Vangalis et al., 2021).

White rot fungi, mainly within Basidiomycota, are well known for their extensive organic compound degradation abilities (Zhuo and Fan, 2021). These species include the most complicated fungi with a wide variety of lifestyles and cell-organization (e.g., mushroom-forming fungi) (Zhuo and Fan, 2021). *Pleurotus ostreatus*, commonly known as oyster mushroom, is one of the most commonly cultivated edible fungi at the global scale, and its wild resources are distributed worldwide. Its hypha and fruiting bodies can be used for environmental remediation, for example, arsenic (As, III) and cadmium (Cd, II) decontamination in water and soil (Li and Xu, 2022). Our previous studies proved ROS to be involved in the defense mechanisms of *P. ostreatus*' to the important pest, *Tyrophagus putrescentiae* (TP), as well as the enhancement of toxic protein and defense metabolite production (Hou et al., 2022; Li et al., 2022; Liu et al., 2023). Here, we discuss the respective roles of NoxA and NoxB in *P. ostreatus*' defense against different biotic and abiotic stresses at several levels, including mycelial growth, mite feeding behavior, gene expression, physiology, and biochemistry metabolism.

## 2 Materials and methods

### 2.1 Strains and culture conditions

*P. ostreatus* strain ACCC50994 was a wild collected strain from Weifang in Shandong Province, and deposited in Agricultural Culture Collection of China (Beijing, China). The TP-sensitive monokaryotic strain 582 (denoted WT hereafter) was derived from spores of ACCC50994 fruit body (Hou et al., 2022). Mycelia were grown onto potato dextrose agar (PDA) medium at 25 ± 1°C and 80% ± 5% relative humidity in complete darkness for 14 d (Qu et al., 2015). The *Escherichia coli* strain DH5α was grown in Luria-Bertani (LB) medium containing 100 g/mL ampicillin at 37°C for plasmid construction and amplification (Liu et al., 2023). An induction medium (IM) containing 100 μg/mL of hygromycin, was used to co-cultivate the *Agrobacterium tumefaciens* strain GV3001 and *P. ostreatus* (Lei et al., 2017). A complete yeast extract medium (CYM) was used to cultivate the Nox transformants of *P. ostreatus* at 25°C under darkness for 10 d (Lei et al., 2017). The vegetative mycelia of the WT strain and Nox transformants were cultivated on PDA medium for 14 d at 25°C for all experiments (Liu et al., 2023).

### 2.2 Mites

Wild populations of *T. putrescentiae* (TP) were collected from the edible fungi, *Auricularia polytricha* in Feng County (Jiangsu, China) in 2012. The mites were bred with fungal mycelium according to previously reported methods (Liu et al., 2023).

### 2.3 *PoNoxA* and *PoNoxB* gene cloning and transformations

Total RNA of the vegetative mycelia was extracted using RNAprep Pure Kit (Tiangen, Beijing, China), and cDNA was reverse transcribed using a First-Stand cDNA Synthesis Kit (Takara, Dalian, China) according to the manufacturer's instructions. Homology searches were conducted on the predicted Noxs using available sequences from the reference sequenced PC15 and PC9 genome (PRJNA670761 and PRJNA81933) and the transcriptome data (PRJNA665192) of *P. ostreatus*. Full length cDNA of the Noxs was amplified by the primer pairs NoxA-1F/NoxA-1R and NoxB-1F/NoxB-1R (Supplementary Table S1) and ligated to the pMD19-T vector (Takara, Dalian, China). It was then transformed into *E. coli* DH5α (Tsingke, Beijing, China) and sequenced. The fungal vector pCAMBIA1303 (Abcam, Shanghai, China) replaced the promoter with a glyceraldehyde-3-phosphate dehydrogenase (GPD) of *P. ostreatus* and was used to construct the overexpression (OE) and RNA interference (RNAi) of *PoNoxA* and *PoNoxB*. The overexpression products corresponded to the Nox coding sequences (without the ATG region) denoted as PoNoxA^OE^ and PoNoxB^OE^, respectively. The RNAi product contained a specific 310 bp fragment for *PoNoxA*, denoted as PoNoxA^RNAi^, and a 307 bp fragment for *PoNoxB*, denoted as PoNoxB^RNAi^. The GPD promoter of *Aspergillus nidulans* and the α-tubulin gene (*PoTub*) of the *P. ostreatus* promoter were used for the construction of the RNAi plasmid to enable the formation of double-stranded RNA. *Agrobacterium tumefaciens*-mediated transformation of *P. ostreatus* was carried out as previously described (Shi et al., 2012). Quantitative real-time PCR (qRT-PCR) was performed for molecular analysis of transformants using a SYBR Premix Ex Taq RT-PCR Kit (Takara, Dalian, China) as previously reported (Liu et al., 2023). The primers were listed in Supplementary Table S1.

### 2.4 Growth analysis

Single colonies of WT and its transformants were transferred to a new CYM plate in a 90-mm Petri dish to observe the growth rate of hyphae at 25 ± 1°C and 80% ± 5% relative humidity (RH) until the entire plate was filled. CYM was supplemented with NaCl (0.6 M) for osmotic stress, Sodium dodecyl sulfate (SDS, 0.014%) for membrane stress, and calcofluor white (600 μg/mL) and congo red (10 mg/mL) individually for cell-wall stress. For the subsequent 15 d, colony' diameter was recorded every 24 h after day 5. Six replicates were used for each strain.

### 2.5 Microscopic examination of mycelia

The hyphal tip shape was observed using an Olympus BX41 microscope (Olympus, Tokyo, Japan). For the evaluation of the morphological changes of the vegetative mycelia in response to mechanical injuries, the tested strain was grown in 5 mL Sedgewick Rafter Counting Chambers with CYM medium (Nuolai, Changzhou, China) for 7 d. The strain was then wounded and photographed at different time points after injury (0, 6, 24, and 48 h), followed by staining with 0.5% lactophenol cotton blue (Yuanye Bio-Technology Co., Shanghai, China) (Cruz-Magalhães et al., 2019).

### 2.6 Hormone and saccharide measurements

A total of 1.0 g of fresh mycelia from the WT, PoNoxA^OE^, PoNoxA^RNAi^, PoNoxB^OE^, and PoNoxB^RNAi^ strains was sampled to analyze the production of hormones and their precursors, including salicylic acid (SA), methyl salicylate (MeSA), jasmonate (JA), 12-oxo phytodienoic acid (OPDA), jasmonoyl-isoleucine (JA-Ile), methyl jasmonate (MeJA), gibberellins (GA1, GA3, GA4, and GA7), indole-3-acetic acid (IAA), IAA-Glu, abscisic acid (ABA), isopentenyladenine (IP), and isopentenyladenosine (IPA), using high-performance liquid chromatography-tandem mass spectrometry (HPLC-MS/MS). These tests were replicated at least four times. The HPLC-MS/MS system was equipped with a SCIEX-6500 Qtrap quadrupole mass spectrometer of AB (Framingham, MA, USA). All samples were pretreated and identified according to previously described methods (Wu et al., 2007; Liu et al., 2023).

For cell wall analysis, the tested strains were incubated in a (potato dextrose broth PDB) medium for 12 days at 25°C. Following this, 10.0 g of mycelia was collected and dried under 65°C using a Yiheng DZF-6050 drier. Monosaccharide and polysaccharide were the dominant cell-wall composition. The amount of the active ingredient contents in total polysaccharide, chitin, β-glucan, and mannose, was quantified using spectrophotometric and HPLC methods (Wu et al., 2014; Li et al., 2020).

### 2.7 RNA-Seq and differential expression analysis

Total RNA was isolated from the fresh mycelium of the WT, PoNoxA^OE^, PoNoxA^RNAi^, PoNoxB^OE^, and PoNoxB^RNAi^ transformants grown for 12 days using the RNA prep Pure Plant Kit (Tiangen, Beijing, China) according to the manufacturer's instructions. Short reads libraries were generated using the NEBNext^®^ Ultra™ RNA Library Prep Kit for Illumina^®^ NovaSeq6000 (NEB, USA) following the instruction manual. Index codes were added to the attribute sequences of each sample (Yan et al., 2018). All measurements were replicated at least three times. Clean reads were mapped onto the *P. ostreatus* PC15 and PC9 reference genomes (PRJNA670761 and PRJNA81933) by third generation PacBio long-read sequencing. The differentially expressed genes (DEGs) were calculated and those with a fragments per kilo base per million (FPKM) expression value of ≥1.5 with a false discovery rate (FDR) of ≤ 0.05 were considered significant.

### 2.8 UPLC-MS-based metabolomics

Each 0.5 g of fresh mycelium from the WT, PoNoxA^OE^, PoNoxA^RNAi^, PoNoxB^OE^, and PoNoxB^RNAi^ transformants was ground into powder using liquid nitrogen in separate polyethylene tubes. Samples were preparated for the mass spectrometry assay as previously reported (Sabino Ferrari et al., 2021). An Ultra Performance Liquid Chromatography (UPLC) system Acquity I-Class PLUS (Waters, USA) coupled with the mass spectrometer Xevo G2-XS QTOF (Waters, USA) was used to analyze the metabolites (Sabino Ferrari et al., 2021). All measurements were replicated six times. Principal component analysis (PCA) was performed using the R (R Core Team) package ropls (version 1.6.3). Differentially expressed metabolites (DEMs) were considered significant in pairwise comparisons for variable importance in the projection (VIP) ≥1.0 and fold change ≥2 or ≤ 0.5.

### 2.9 Bioassays for Noxs-induced resistance against *T. putrescentiae*

Host choice, population growth, and egg hatchability of the *PoNoxA* and *PoNoxB* transformants were tested for the resistance against *T. putrescentiae*. For the host choice test, a 1.0 cm block of 10 day-grown mycelium (containing PDA medium) was randomly placed in 150 mm Petri dishes divided into four quadrants. For five different mycelium blocks, the distance between them was equal and the center of the Petri dish. Mites were released onto the center of the Petri dish (100 adults per Petri dish) and the dish was subsequently covered. These mites were starved for 24 h, and then the trapping rate of each strain was counted (Hou et al., 2022). The experiments were conducted at 26 ± 1°C and 80% ± 5% RH. To determine egg hatchability and mite development duration, 30 newly eggs of *T. putrescentia* were placed on each transformant, and mite's development traits were daily recorded in 21 d (Liu et al., 2023). The WT strain was used as the control. Six biological replicates were set up for all experiments under 28°C and 80% RH. To provide evidence that TP indeed did feed on transformants, we collected photographs at 0, 6, 24, and 48 h after placing the mites on the mycelium and weighed the loss of fungus mycelium after the mites fed for 24 h.

### 2.10 Statistical analysis

Data analysis and graphs were prepared using Microsoft Excel (version 2016, Microsoft Corp.). Significant differences were analyzed using one-way analysis of variance (ANOVA) and Duncan's multiple range test. Statistical analysis was performed using IBM SPSS 21.0 (IBM, Somers, USA).

## 3 Results

### 3.1 *PoNoxA* and *PoNoxB* differentially regulate mycelial formation and branches of *P. ostreatus*

Noxs have been demonstrated as necessary for a number of physiological functions and cellular differentiations in fungi, including the formation of sexual fruiting bodies, and the germination of ascospores, hyphal defense, and hyphal growth (Marschall and Tudzynski, 2016; Wang et al., 2022). In this study, we monitored the change in mycelial formation and branches caused by the two NADPH oxidases of *P. ostreatus*. The *PoNoxA* and *PoNoxB* transformants were randomly selected and transferred to PDA plates and sub-cultured five times for hereditary stability. According to the results of PCR and qRT-PCR assays, we chose the transformants N1O-13 strain as the PoNoxA^OE^ strain, N1i-10 strain as the PoNoxA^RNAi^ strain, N2O-2 strain as the PoNoxB^OE^ strain, and N2i-11 strain as the PoNoxB^RNAi^ strain in this study (Supplementary Figure S1). Microscopic observations revealed that WT, *PoNoxA* overexpression and *PoNox* knockdown strains had development of smooth, thin, branching hyphae (Supplementary Figures S2A–C, E), whereas *PoNoxB* overexpression strains showed a relatively different appearance, that was, rough and thick basal hyphae and curved branching mycelium (Supplementary Figures S2D, F). Three distinct lateral branching trends were observed, namely, dichotomous, trichotomous, and random branching (Supplementary Figure S2). These results showed that *PoNoxB* is principally required for hyphal formation of the multicellular structure, while *PoNoxA* regulates hyphal tip growth. Branch formation is an unquestionable characteristic of fungal hyphae that underlies the ability of the fungi to form filamentous mycelia (Harris, 2008). Numerous mutations that enhance apical branching and knobby appearing hyphae, also known as dichotomous branching or tip splitting, have been described in fungi such as *A. nidulans, A. niger, Neurospora crassa, T. reesei*, and Basidiomycetes *Coprinopsis* species (Kwon et al., 2013; Sakamoto et al., 2018; Fitz et al., 2019).

### 3.2 *PoNoxA* regulates *P. ostreatus* morphogenesis under diverse stress conditions

**Figure 1:**
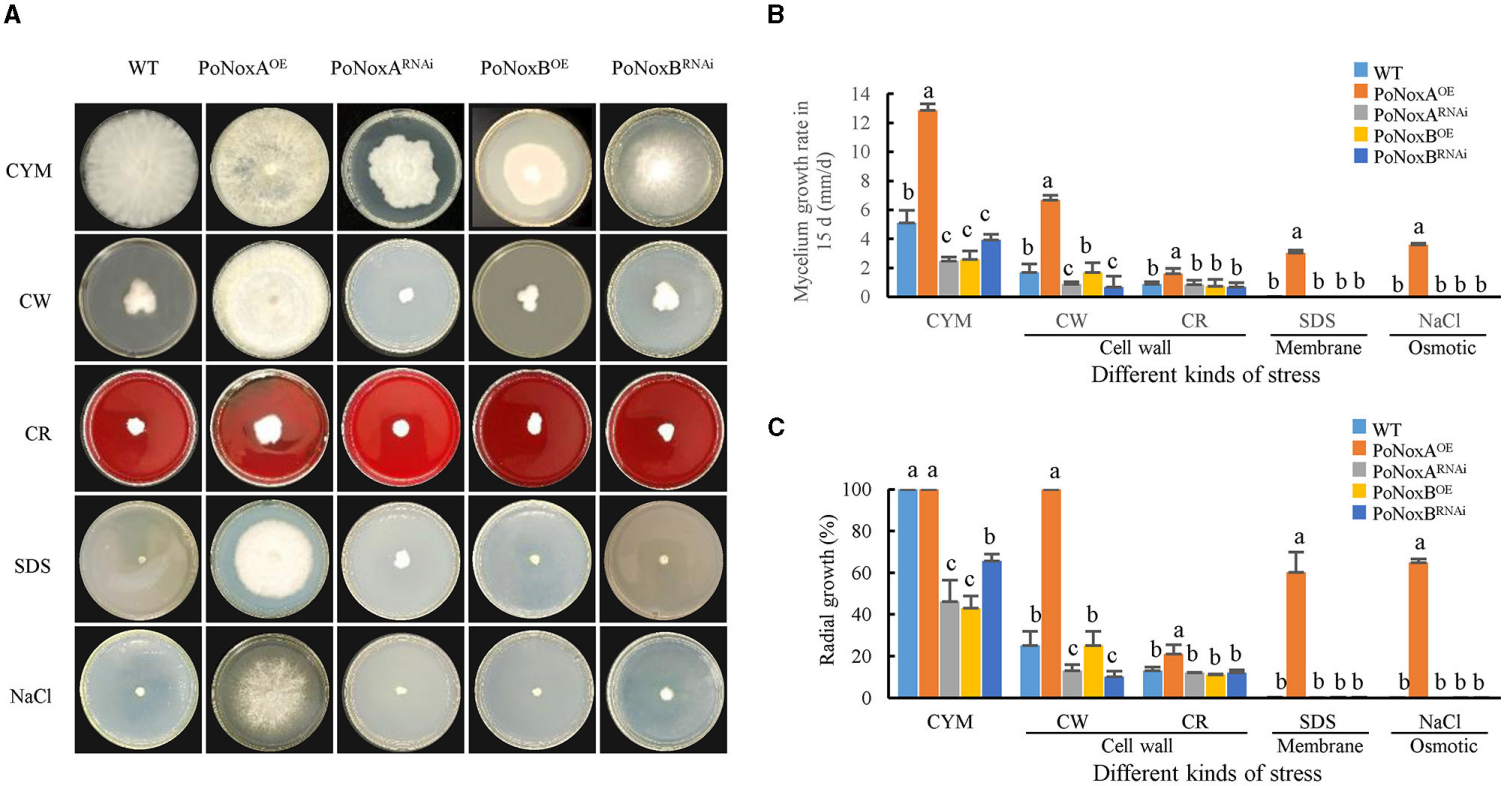
Stress response of *P. ostreatus* Nox mutants. **(A)** The WT and four Nox mutants were inoculated to the center of CYM plates at 25°C for 15 d, supplemented with NaCl (0.6 M NaCl) for osmotic stress, SDS (0.014% SDS) for membrane stress, and CW (600 μg/mL calcofluor white) and CR (10 mg/mL congo red) individually for cell-wall stress. **(B)** The graphs display the relative values of mycelial growth rate in 15 d, and **(C)** The graphs display the mycelial radial growth (%) in 15 d.

The WT, PoNoxA^OE^, PoNoxA^RNAi^, PoNoxB^OE^, and PoNoxB^RNAi^ strains were evaluated for growth and conidiation under osmotic, membrane, and the cell-wall stress conditions to confirm the significant phenotypic effect of single mutations on the two NADPH oxidase genes of *P. ostreatus*. The PoNoxA^OE^ strains displayed distinct trends in colony morphological changes (Figure 1A). By contrast, the *PoNoxB* mutant exhibited colony morphologies that changed almost consistently with those of the WT in all treatments, except for growth rate on CYM (Figures 1A, B). The growth rates of the WT, *PoNoxA* and *PoNoxB* transformants were also compared under a 15-day cultivation treatment. Mycelial growth was greatly impeded when these two genes were knocked down, as shown in Figure 1B (*p*-value < 0.01). This indicates that *PoNoxA* and *PoNoxB* play key roles in the vegetative growth of *P. ostreatus*. The mycelial radial growth of all knockdown strains revealed a quantitative decline relative to 30%-50% of the WT strain on the CYM medium (Figure 1C). However, the effect of *PoNoxA* and *PoNoxB* overexpression on mycelial growth was reversed. PoNoxA^OE^ reached 12.86 mm/d, which was 2.5, 5.2, and 5.0 times that of WT, PoNoxA^RNAi^, and PoNoxB^OE^, respectively, taking only 7 days rather than 15 d (WT) to fill the entire plate on the CYM medium (Figure 1B). *PoNoxA* and *PoNoxB* were involved in different cellular functions during the vegetative growth stage of *P. ostreatus*. For example, unlike *PoNoxB* and *PoNoxA* was observed to positively regulate hyphal growth.

By cultivating these strains on plates under varying stress conditions, we tested their resistance to osmotic, membrane and cell-wall stress. Consistent with the morphogenesis observations, the *PoNoxB* transformants and WT showed no significant differences in mycelial radial growth and growth rate under the four stress conditions analyzed, and the mycelium appeared to stop growing (Figures 1A, B). This finding is in agreement with that reported by Cruz-Magalhães et al. (2019), who found that the Δnox2/B mutant in *Trichoderma atroviride* exhibited colony morphologies highly similar to those of the WT in all treatments (Cruz-Magalhães et al., 2019). In this study, we observed that *PoNoxA* positively regulated mycelial morphogenesis to increase resistance to these additional environmental pressures. Over-expression of *PoNoxA* exhibited a high tolerance to the osmotic stress of 0.6 M NaCl, cell wall stress of 600 μg/mL calcofluor white and cell membrane of 20% SDS. To permit growth under high osmotic conditions with 0.6 M KCl, the hyphae of *N. crassa* reduced the pressure by increasing the elongation rate widths (Fukuda et al., 2021). However, all strains were sensitive to 10 mg/mL congo red (CR) by greatly reducing mycelial mass by >70% (Figure 1C). This higher sensitivity to CR is also seen in other fungi, for example, CR caused a 60% decline in the mycelial mass of Nox mutants.

### 3.3 *PoNoxB* increases the cell wall composition of saccharides

**Figure 2:**
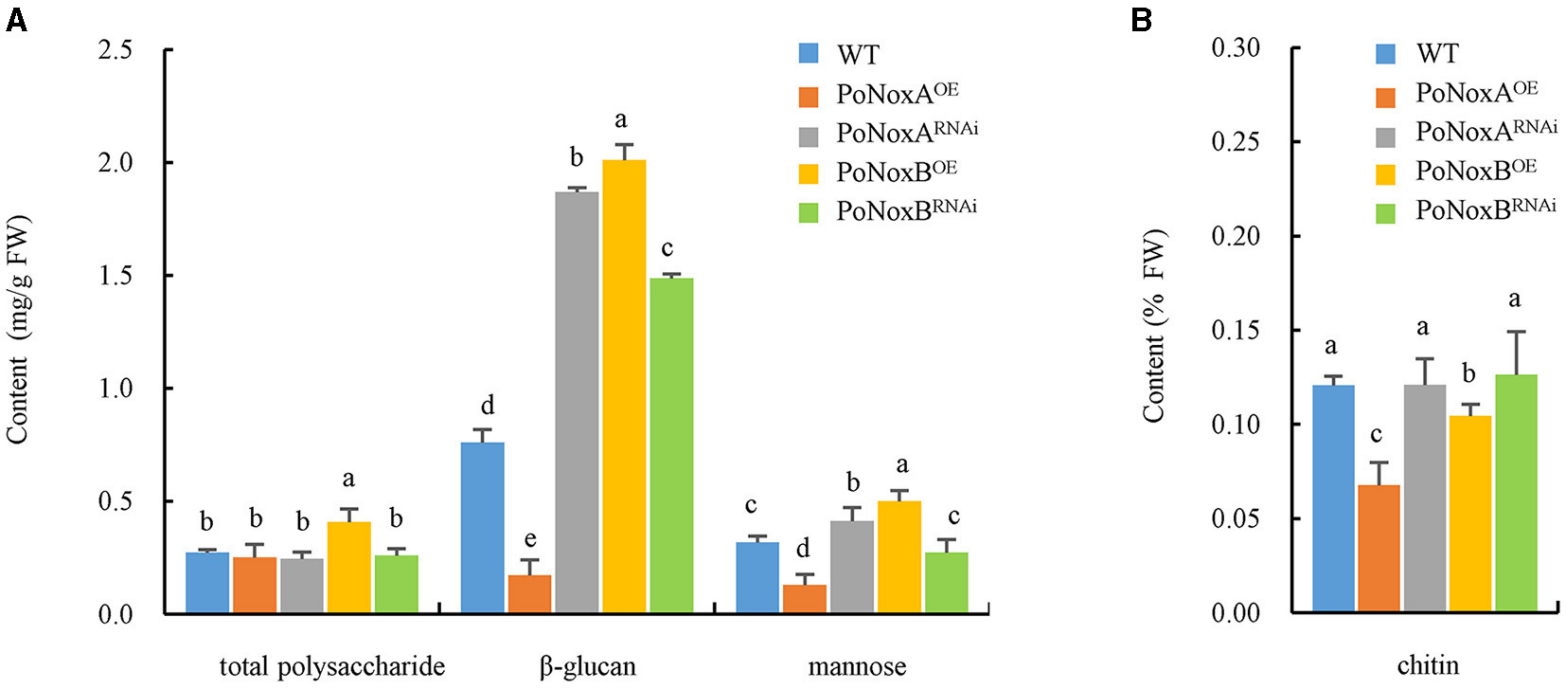
The content of total polysaccharide, β-glucan, mannose and chitin in the cell-wall of *P. ostreatus* Nox mutants. **(A)** The content of total polysaccharide, β-glucan, and mannose in *PoNoxA* and *PoNoxB* transformants. **(B)** The content of chitin in *PoNoxA* and *PoNoxB* transformants. Letters indicate significant differences among strains (*p* < 0.05, Duncan's test).

Fungal walls are complex structures mainly composed of polysaccharides (>90%), such as glucans, chitin and glycoprotein, which are embedded in the cell and linked to respond to abiotic and biotic stresses (Latgé, 2007; Huang et al., 2023). Therefore, we investigated the impact of Noxs on cell wall composition in the vegetative mycelia of *P. ostreatus*. The over-expression of *PoNoxB* significantly increased the content of total polysaccharide (0.408 mg/g), β-glucan (2.010 mg/g), and mannose (0.500 mg/g) by 49%, 164%, and 57.2% when compared with WT, respectively, as shown in Figure 2A (*p* < 0.05). However, over-expression of *PoNoxA* did not increase these saccharides, yet its knockdown strain increased β-glucan and mannose contents by 146% and 30% (Figure 2A). No significant increases were observed in the chitin in the *PoNoxA* and *PoNoxB* mutations (Figure 2B).

### 3.4 *PoNoxA* and *PoNoxB* diversely modulate hormonal levels

**Figure 3:**
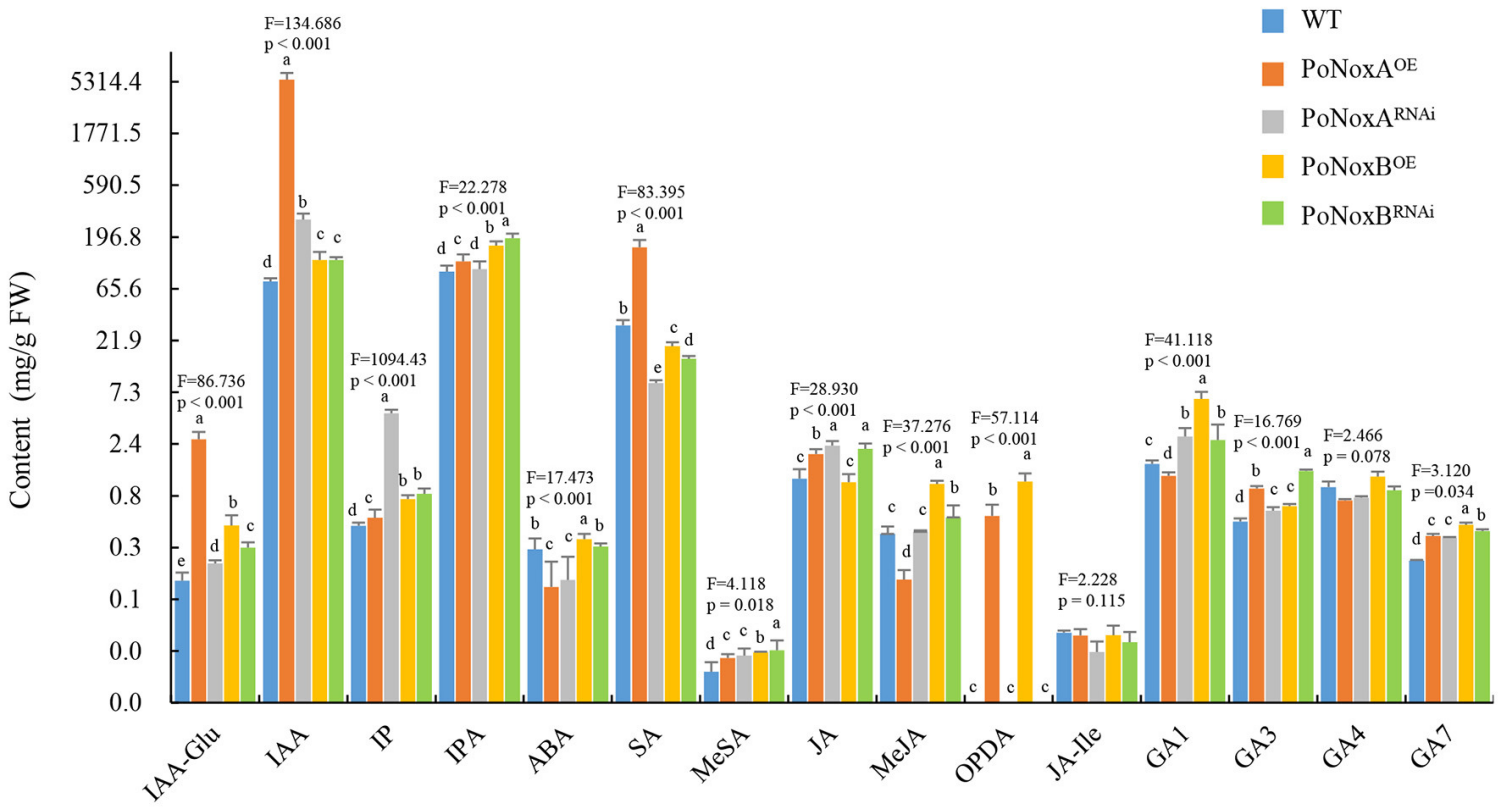
The changes of hormone levels in the vegetative mycelium of *P. ostreatus* Nox mutants detected by HPLC-MS/MS. The different hormone levels include the production of indole-3-acetic acid (IAA), IAA-Glu, isopentenyladenine (IP), isopentenyladenosine (IPA), abscisic acid (ABA), salicylic acid (SA), methyl salicylate (MeSA), jasmonate (JA), 12-oxo phytodienoic acid (OPDA), jasmonoyl-isoleucine (JA-Ile), methyl jasmonate (MeJA), and gibberellins (GA1, GA3, GA4, and GA7). Letters indicate significant differences among strains (*p* < 0.05, Duncan's test).

**Figure 4:**
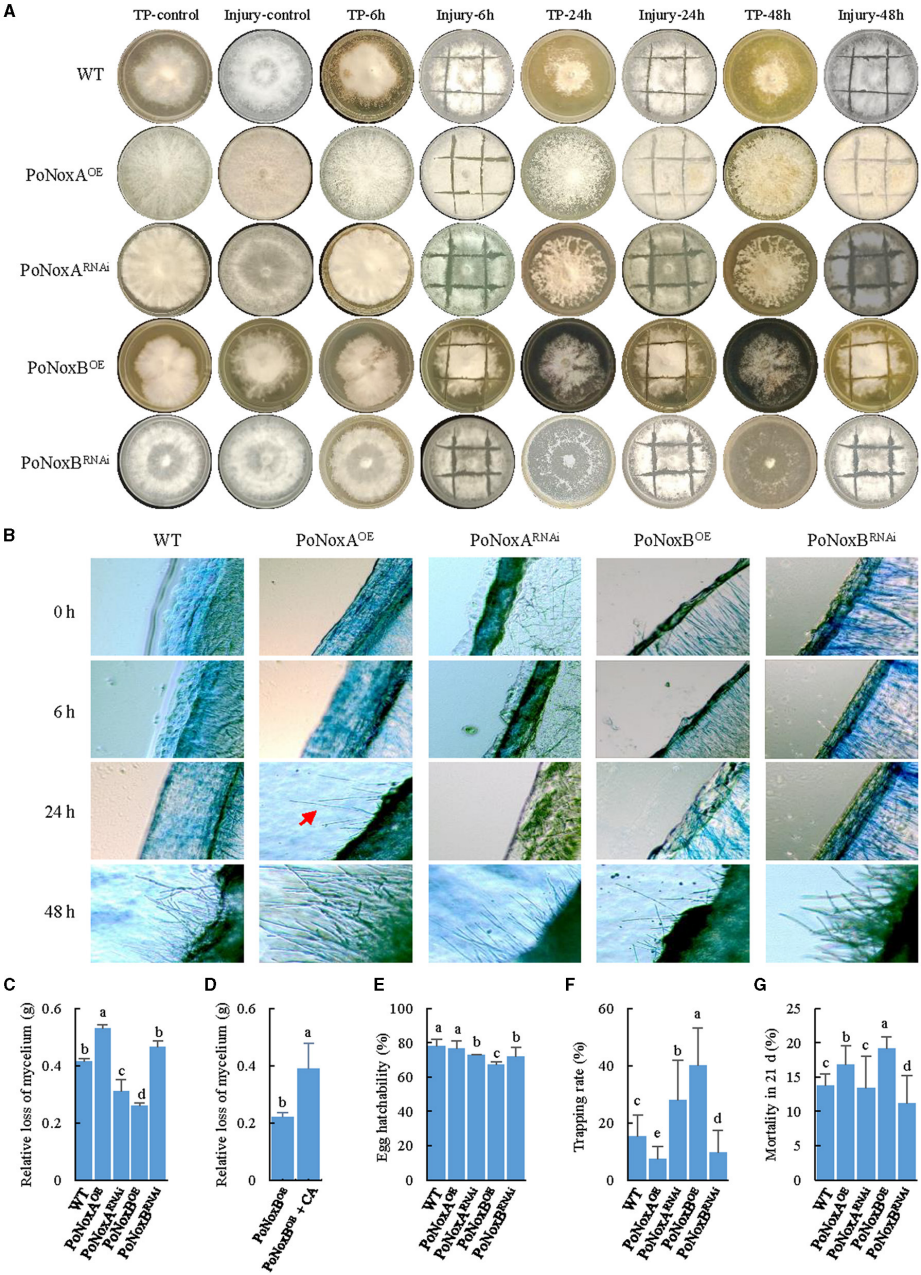
The mechanical injury and fungivory response of *P. ostreatus* Nox mutants. **(A)** The macroscopic observation of the mechanical injury and *T. putrescentiae* feeding response after different treatment times (6, 24, and 48 h). **(B)** Microscopic changes of the vegetative mycelium observed upon injury. Mycelial samples of the WT and mutants were stained with 0.5% lactophenol cotton blue. **(C–G)** Bioassays for Nox-induced resistance against *T. putrescentiae*. The impact of fungivory on the relative loss of mycelium **(C)**, egg hatchability **(E)**, the host choice **(F)**, and mite mortality **(G)** in 21d. **(D)** Quantification of mycelian loss between the PoNoxB^OE^ and PoNoxB^OE^ added caspofungin acetate. Letters indicate significant differences among strains (*p* < 0.05, Duncan's test).

Noxs were reported to be involved in GAs change in *G. lucidum*, however, the role of Noxs on fungal metabolism of endogenous hormones remain largely unexplored (Zhu et al., 2022). Much work has been made recently in understanding the potential effects of exogenous hormones on fungal growth, development, and secondary metabolism (Mu et al., 2014; Liu et al., 2018). To examine the effect of the Noxs on the vegetative mycelia morphogenesis, growth, and innate immune systemic response in *P. ostreatus*, we measured the hormonal levels of IAA, IAA-Glu, IP, IPA, ABA, JA, JA-Ile, OPDA, MeJA, SA, MeSA, and GAs in the transformants strains. *PoNoxA* and *PoNoxB* may regulate different hormonal levels in the vegetative mycelia of *P. ostreatus* (Figure 3). The production of IAA, IAA-Glu, IP, MeSA, GA3, and GA7 significantly increased in the *PoNoxA* and *PoNoxB* mutants, while IAA and IAA-Glu levels were greatly enhanced in the PoNoxA^OE^ strain (nearly 21.5 and 71.4 times compared with WT, respectively; Figure 4). However, silencing of PoNoxA also leads to increases in IAA and IAA-Glu (nearly 1.5 and 3.7 times, respectively, compared with WT). The biosynthesis of OPDA was enhanced by over-expression of both *PoNoxA* and *PoNoxB*. Moreover, the PoNoxB^OE^ strain induced the greatest increase in the MeJA, OPDA, and GA1 levels compared with the other strains (Figure 3). However, no significant differences were observed in the production of JA-Ile and GA4 among these strains.

### 3.5 Altered expression of Noxs significantly affects the innate immune systemic response of *P. ostreatus* to mechanical injury and fungivory

We described different response between mechanical injury and TP feeding using macroscopic and microscopic observations. Based on both observation types, the first morphological change in the *P. ostreatus* colonies was observed 48 h after injury, except for the *PoNoxA* over-expression strain, which formed aerial mycelium after 24 h (Figures 4A, B). Notably, the morphological changes were obvious after TP feeding for 6 h. As shown in Figure 4A, 24 h after fungivory, white mycelia were significantly reduced and left numerous holes on the mediums. The impact of fungivory on the relative loss of mycelium was significantly different among these strains, with a loss range of 0.26–0.53 g after 24 h (*F* = 135.899, *p* < 0.001; Figure 4C). Interestingly, although TP consumed the maximum amount of mycelium, after 48 h, the formation of the fast-growing “new” hyphae covered many previous holes after TP feeding (Figure 4A). We used caspofungin acetate (CA) as an inhibitor of fungal cell wall biosynthesis to verify whether the resistance of *P. ostreatus* against TP due to *PoNoxB* enhances the polysaccharide content of cell wall. The *PoNoxB* over-expression strain was put 0.05 mg/L caspofungin acetate on the mycelia after the strain was inoculated for 7 d on the CYM plate, and then the strain was continued to culture for 21 d. Mycelium loss was observed to differ significantly between the PoNoxB^OE^ and PoNoxB^OE^ added CA strains (*F* = 47.355, *p* < 0.001) following 24 h of TP feeding (Figure 4D).

Host choice, population growth, egg hatchability, and mortality were also significantly different in the resistance of these strains against TP (Figures 4E–G, Table 1). For host preference, compared with the WT strain, TP induced a significant addiction for the strains PoNoxB^OE^ and PoNoxA^RNAi^ (*F* = 11.225, *p* < 0.001), while it demonstrated a lower trapping rate for the strains PoNoxA^OE^ and PoNoxB^RNAi^ (Figure 4F). The total egg-to-adult development time varied from 10.9 ± 0.3 d (reared with PoNoxA^RNAi^) to 13.6 ± 0.8 d (reared with PoNoxA^OE^; Table 1), and the mortality varied from 10.9% ± 0.1% (reared with PoNoxB^RNAi^) to 19.9% ± 0.0% (reared with PoNoxB^OE^) (Figure 4G). We also observed the resistance of *PoNoxB* to mite feeding with the lowest egg hatchability and relative loss of mycelium and the highest mortality (Figures 4C, E, Table 1).

**Table 1:** The developmental period of *T. putrescentiae* on different strains of *P. ostreatus*.

**Strain**	Egg^**^	**Larvae**	Protonymph^*^	Tritonymph^*^	Total^**^
WT	4.2 ± 0.1b	2.4 ± 0.4	2.6 ± 1.0c	3.1 ± 1.0a	12.4 ± 0.8c
PoNoxA^OE^	4.6 ± 0.0a	2.4 ± 0.6	4.0 ± 0.1a	2.6 ± 0.4b	13.6 ± 0.8a
PoNoxA^RNAi^	3.8 ± 0.2c	2.7 ± 0.3	2.4 ± 0.4c	2.0 ± 0.0c	10.9 ± 0.3d
PoNoxB^OE^	4.2 ± 0.0b	2.6 ± 0.4	4.0 ± 0.3a	2.2 ± 0.0c	13.0 ± 0.2b
PoNoxB^RNAi^	4.5 ± 0.0a	2.8 ± 0.2	3.5 ± 1.1b	2.1 ± 0.1c	12.8 ± 0.8bc

Values are presented as mean ± SE. Means within a column followed by the same letter are not significantly different (LSD test: *p* < 0.05). Asterisk indicates that there are statistically significant differences between five *Pleurotus ostreatus* hosts at each parameter level (^*^p < 0.05 and ^**^p < 0.01).

### 3.6 Roles of *PoNoxA* and *PoNoxB* in the regulation of gene expression and metabolite production in *P. ostreatus*

**Figure 5:**
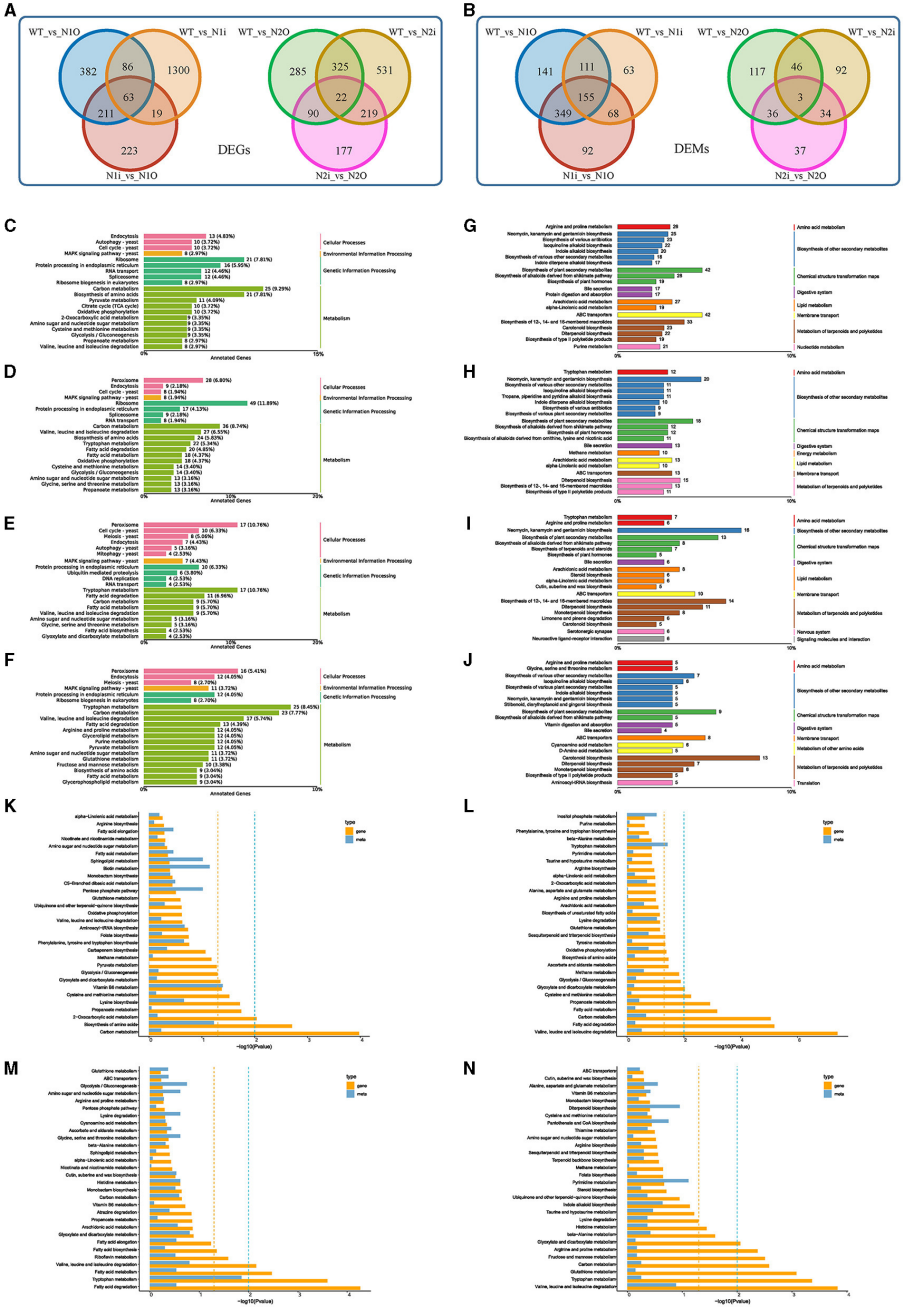
Expression analysis of genes, metabolites after the *PoNoxA* and *PoNoxB* overexpression and knockdown. **(A, B)** The Venn diagram shows the overlap between sets of differentially expressed genes (DEGs) and metabolites (DEMs) among the WT, *PoNoxA* and *PoNoxB* mutants, respectively. **(C–F)** Summarized main KEGG pathways of DEGs after *PoNoxA* overexpression (N1O), *PoNoxA* knockdown (N1i), *PoNoxB* overexpression (N2O), and *PoNoxB* knockdown (N2i), respectively. **(G–J)** Summarized main KEGG pathways of DEMs after *PoNoxA* overexpression (N1O), *PoNoxA* knockdown (N1i), *PoNoxB* overexpression (N2O), and *PoNoxB* knockdown (N2i), respectively. **(K–N)** Metabolome and transcriptome analysis of *PoNoxA* overexpression (N1O), *PoNoxA* knockdown (N1i), *PoNoxB* overexpression (N2O), and *PoNoxB* knockdown (N2i), respectively, by KEGG enrichment analysis *p*-value histogram of the DEGs and DAMs.

To comprehensively investigate the intrinsic molecular mechanism of *PoNoxA* and *PoNoxB* and the effects of hyphal growth, glycan deposition, stress response, and innate immunity, we performed RNA-Seq (PRJNA1000455) and metabolomic analysis of the WT and four mutant strains. After *PoNoxA* overexpression and knockdown, the identified DEGs (742 and 1,468, respectively) and DEMs (756 and 397, respectively) were compared with those of WT strain (Figure 5A, Supplementary Table S2). Furthermore, 516 DEGs and 664 DEMs were found in the two *PoNoxA* mutants. The DEGs in the *PoNoxA* mutants were related to metabolism (e.g., carbon metabolism and amino acids biosynthesis) and ribosome, and were the most significantly enriched pathway in the KEGG analysis. By contrast, the majority of the DEMs were associated with ABC transporters, metabolism of terpenoids and polyketides (e.g., biosynthesis of 12-, 14-, and 16-membered macrolides and biosynthesis of type II polyketide products), isoquinoline alkaloid biosynthesis, and biosynthesis of plant secondary metabolites (Figures 5C, D, G, H).

Combined with the metabolomics and transcriptomics results of the WT strain, the KEGG pathways of vitamin B6 metabolism were both significantly enriched in the PoNoxA^OE^ strain (Figure 5K). Cell growth and death were associated with 16 DEGs in cell cycle-yeast and meiosis-yeast (e.g., anaphase-promoting complex activator Cdc20 and cell cycle serine/threonine-protein kinase Cdc5). In addition, signal transduction was associated with 9 DEGs (e.g., nuclear proteasome tether protein Cut8 [gene-PC9H_003564], general transcriptional corepressor [gene-PC9H_004144], catalase 1 [gene-PC9H_005726], and Pho guanine nucleotide exchange factor Scd1 [gene-PC9H_007269]) in the MAPK signaling pathway-yeast and phosphatidylinositol signaling system, which were enriched in the transcriptomics of PoNoxA^OE^ strain (Figure 5C, Supplementary Table S2). MAPK and ROS signaling pathways as early signaling events play a key role in the innate immune recognition of filamentous fungi (Medina-Castellanos et al., 2014). NoxA-dependent ROS in *A. nidulans* are essential for sexual differentiation regulated by the MAPK kinase SakA (Lara-Ortíz et al., 2003; Lara-Rojas et al., 2011). Moreover, gene-PC9H_004144 was identified as highly homologous to *Ssn6* from the fungi *Saccharomyces cerevisiae* and *Candida albicans*, which regulated organisms to develop true hyphae, extensive filamentation and virulence (Hwang et al., 2003). In yeast, Cut8 was reported to exhibit a repair function of the proteasome under DNA damage (Takeda et al., 2011). Scd1, a guanine nucleotide exchange factor of the Rho-family GTPase Cdc42, is involved in the coordination of cell polarity regulation, cytokinesis, and response to environmental stresses (Tay et al., 2018; Salat-Canela et al., 2021). Our previous findings reveal that the JA signaling pathways and bioactive terpenoid metabolism mediate the direct resistance of *P. ostreatus* against TP (Li et al., 2018, 2022). The expression of the methyltransferase Lae1 (gene-PC9H_000701) orthologous *Aspergillus* spp. LaeA protein was significantly upregulated in PoNoxA^OE^ strain, LaeA is known to regulate secondary metabolic gene clusters including sterigmatocystin (an insecticidal mycotoxin), penicillin, and terrequinone A pathways (Bok et al., 2005; Perrin et al., 2007). Moreover, three major structural moieties of anti-mite toxin-avermectins were produced in PoNoxA^OE^ strain (A1a, A1b, and A2b). However, the function of the LaeA protein has not been reported on *P. ostreatus*. Whether the formation of these anti-mite compounds is correlated with LaeA is still unclear. In *A. nidulans*, larval grazing of *Drosophila melanogaster* triggered the expression of *LaeA* (Caballero Ortiz et al., 2013). Considering the findings of the present study, *PoNoxA* is likely to positively regulate hyphal tip growth, polarized growth, stress response and innate immunity. Another most important role of *PoNoxA* was the regulation of laccase expression. The important applications of laccases are in bioremediation and other various industrial and biotechnological areas (Li and Xu, 2022).

Following *PoNoxB* overexpression and knockdown, the identified DEGs (722 and 1,097, respectively) and DEMS (202 and 175, respectively) were compared with the WT strain, with 347 DEGs and 49 DEMs found in the two treatments (Figure 5B, Supplementary Table S2). The DEGs in the *PoNoxB* mutants were concentrated in metabolism (e.g., fatty acid degradation, tryptophan metabolism and valine, leucine, and isoleucine degradation), while more than 30% of the DEMs were associated with chemical structure transformation maps, terpenoid and polyketide metabolism, and biosynthesis of other secondary metabolites (Figures 5E, F, I, J). Furthermore, in the production of secondary metabolites, particularly terpenoid and polyketide metabolism, *PoNoxB* mutants enhanced the production of bioactive antibiotics or toxalbumins (e.g., GAs, aconitine, zeatin, rifamycin B, microcystin LR, and dihydrogranaticin). Note that 17 DEGs were identified in the peroxisome pathway after *PoNoxB* overexpression, including catalase Cat1 and Cat2, NADH pyrophosphatase Npy1, and 3-ketoacyl-CoA thiolase Pot1. Based on the enhancement of polysaccharide production after *PoNoxB* overexpression, we analyzed the differences in genes and metabolites in polysaccharide metabolism. Seven DEGs and nine DEMs were observed to be upregulated in polysaccharide metabolism, including two genes (gene-PC9H_002050 and gene-PC9H_006828) annotated to the cell wall/membrane /envelope biogenesis, β-glucan synthesis and β-1, 3-glucan synthase FKS1, three genes in cell-wall integrity (CWI) pathway, one compound in mannose type *O*-glycan biosynthesis, and eight compounds in fructose and mannose metabolism. These results provide evidence that *PoNoxB* may be a signal component of inducible polysaccharide metabolism and *P. ostreatus* tolerance against biotic stress.

To leverage both the transcriptomics and metabolomics results and investigate the molecular mechanisms for the fungal developmental processes and external stress responses, we performed a comparative analysis of the different effects between the *PoNoxA* and *PoNoxB* mutants (Figures 5K–N, Supplementary Figure S3). The results indicate that the *PoNoxA* and *PoNoxB* mutants affect differentially abundant genes and metabolites (Supplementary Figures S3A, B). Moreover, in the results of KEGG analysis revealed 92 DEGs and 212 DEMs shared by the transcriptomics and metabolomics of the overexpression of *PoNoxA* and *PoNoxB* (Supplementary Figure S3C). The most significantly enriched pathway in the KEGG analysis was related to the biosynthesis of amino acids [e.g., L-2-aminoadipate, 6-semialdehyde and (2R, 3R)-2, 3-dihydroxy-3-methylpentanoate], carbon metabolism (e.g., glycolate, glyceric acid, and D-glycerate3-phosphate), 2-oxocarboxylic acid metabolism and folate biosynthesis (Supplementary Figure S3).

## 4 Discussion

Fungi are eukaryotic microorganisms that generate simple tube-like hyphae that can differentiate into more complex morphological structures and diverse cell types. The complex multicellular entities of fungi are reproducing individuals that perform multiple functions, such as nutrient acquisition, mating, pathogenicity, immune defense, and biodegradation of environmental pollutants (Virágh et al., 2022). One of the most important but least understood elements of fungal cell biology is the ability of rapidly expanding hyphae to generate new polarity axes that result in the creation of branches. Branching is essential for the growth of mycelial colonies and is considered as key in fungal interactions with other organisms (Fukuda et al., 2021).

In this study, we revealed that the two Noxs (*PoNoxA* and *PoNoxB*) of *P. ostreatus* were responsible for hyphal tip growth, branching and the polysaccharide composition of the cell wall. Numerous mutations of Noxs have been principally responsible for spore germination, apical dominance, and induction of abundant hyphal branching. In the majority of filamentous fungi, particularly in *Ascomycota* spp., both NoxA and NoxB induce the formation of multicellular structures (Cano-Domínguez et al., 2008). In *G. lucidum*, NoxA, and NoxB are both required for ROS generation and hyphal branching (Mu et al., 2014). In *P. ostreatus*, the present study showed that PoNoxA positivity regulated hyphal tip growth and that PoNoxB was mainly required for hyphal formation and the cell wall composition of saccharides. Moreover, Nox mutants (ΔnoxR, ΔnoxA/1, and ΔnoxB/2) are affected by the regulation of fungal development under different stress conditions (Mu et al., 2014; Cruz-Magalhães et al., 2019; Vangalis et al., 2021). We also characterized a complex regulatory network in different biotic and abiotic stress responses at the transcriptome, cellular, and physiological levels. This study found that PoNoxA and PoNoxB were observed to play different roles across multiple regulatory programs. PoNoxA played an essential role in SDS, CW and CR stress tolerance, SA metabolism and damage repair, while PoNoxB enhanced the biosynthesis of MeJA, OPDA, and GA1. Both two genes were involved in IAA, IAA-Glu, IP, and MeSA biosynthesis. The silencing and overexpression of PoNoxA both lead to increases in IAA and IAA-Glu; such puzzling results need to be studied further. In recent years, studies have found that Noxs (also known as Rbohs in plants) are frequently induced by diverse phytohormones and coordinately modulate plant development and stress tolerance (Sun et al., 2021). In *N. crassa*, the inactivation of catalase-3 results in enhanced protein oxidation, higher carotene levels, hyphal adhesion and higher amounts of aerial hyphae and conidia (Aguirre et al., 2005).

The Nox mutants were observed to share several functions in primary and secondary metabolism, signal transmission, and toxic protein and defense metabolite production (Hernández-Oñate et al., 2012; Caballero Ortiz et al., 2013). By using an insect-fungus competition model system, such as *A. nidulans* and *D. melanogaster*, the significant role of global secondary metabolite regulation was found for some fungal genes, as *VeA, HdaA* and *Ppo* directly or indirectly influenced insect development (Trienens and Rohlfs, 2012). However, distinct and conserved roles were also identified. Here, we found three major structural moieties of anti-mite toxin produced in the PoNoxA^OE^ strain. In recent studies, LaeA-like genes in *P. ostreatus* regulate fungal growth, cellulase activity, intracellular ROS signaling, and secondary metabolism biosynthesis (e.g., polysaccharide) (Zhang et al., 2022). However, it remains unclear whether PoNoxA and PoLaeA unction together to control these toxin metabolisms by regulating intracellular ROS signaling. In particular, PoNoxA was strongly involved in the cell growth and death pathway with 16 DEGs in cell cycle-yeast and meiosis-yeast, as well as signal transduction with 9 DEGs in MAPK signaling pathway-yeast and the phosphatidylinositol signaling system. Similarly, PoNoxB regulated the production of bioactive antibiotics and toxalbumins (e.g., GAs, aconitine, zeatin, rifamycin B, microcystin LR, and dihydrogranaticin).

In summary, we revealed that the two Noxs (PoNoxA and PoNoxB) of *P. ostreatus* were responsible for hyphal tip growth, branching and polysaccharide composition of the cell wall. We also characterized a complex regulatory network in different biotic and abiotic stress responses at the transcriptome, cellular, and physiological levels. PoNoxA and PoNoxB were observed to play different roles across multiple regulatory programs. For example, *PoNoxA* positivity regulated hyphal tip growth and *PoNoxB* was mainly required for hyphal formation and the cell wall composition of saccharides, while *PoNoxA* played an essential role in SDS, CW and CR stress tolerance; SA metabolism and damage repair. However, the two Noxs, also exhibited the partial overlapping of several functions in the JA and GA metabolism pathways, compound metabolism, signal transmission, and mushroom defense against TP. Our findings suggest that the oyster mushroom appears to respond to different stresses under a delicate growth-defense balance by adjusting the ROS signal regulated by the Nox complex.

## Data availability statement

The datasets presented in this study can be found in online repositories. The names of the repository/repositories and accession number(s) can be found in the article/Supplementary material.

## Ethics statement

The manuscript presents research on animals that do not require ethical approval for their study.

## Author contributions

HL: Writing – original draft, Methodology. JZ: Writing – original draft, Visualization, Software, Data curation. ZL: Writing – review & editing, Methodology. PX: Writing – review & editing, Formal analysis. LM: Writing – review & editing, Data curation. YZ: Writing – review & editing. SQ: Writing – review & editing, Funding acquisition, Conceptualization. XW: Writing – review & editing, Project administration.
