# Supplementary material for: Contrasting effects of NADPH oxidases on the fungal hyphae growth and immune responses in Pleurotus ostreatus
Source: Front Microbiol. 2024 Jun 19;15:1387643. doi: 10.3389/fmicb.2024.1387643 (PMC11220167; doi:10.3389/fmicb.2024.1387643)
Supplement: Supplementary file 1 [file Data_Sheet_1.DOCX]

Table S1 Primers used in this study

| **NO.** | **Gene name** | **Primer name** | **Primer sequence (5' to 3')** | **Analysis** |
| --- | --- | --- | --- | --- |
| 1 | *β*-*Tubulin* | Tub-Q1F | AGGCTTTCTTGCATTGGTACACGC | qRT-PCR for *β-tubulin* expression |
|  |  | Tub-Q1R | TATTCGCCTTCTTCCTCATCGGCA |  |
| 2 | *PoNoxA* | NoxA-1F | ATGGGCTTCAAGAGCTGGTTC | Full-length cDNA cloning of *PoNoxA* |
|  |  | NoxA-1R | TTAGAAATGTTCCTTCGCAA |  |
|  |  | NoxA-Q1F | CTTCCCAGCCGATGAGAATATC | qRT-PCR for *PoNoxA* expression |
|  |  | NoxA-Q1R | AGTTGACGTAGTGAGCTGTTG |  |
| 3 | *PoNoxB* | NoxB-1F | ATGATTAACGAAGGAGGGA | Full-length cDNA cloning of *PoNoxB* |
|  |  | NoxB-1R | TTAGAAGTTCTCCTTTCCGA |  |
|  |  | NoxB-Q1F | TTGCCCTGAGGTATCCTACT | qRT-PCR for *PoNoxB* expression |
|  |  | NoxB-Q1R | GTCACCGCAAACACGAATG |  |
| 4 | *HygB* | Hyg-Q1F | CCGCAAGGAATCGGTCAATA | qRT-PCR for hygromycin gene expression |
|  |  | Hyg-Q1R | GGTGTCGTCCATCACAGTTT |  |
