# Supplementary material for: Contrasting effects of NADPH oxidases on the fungal hyphae growth and immune responses in Pleurotus ostreatus
Source: Front Microbiol. 2024 Jun 19;15:1387643. doi: 10.3389/fmicb.2024.1387643 (PMC11220167; doi:10.3389/fmicb.2024.1387643)
Supplement: Supplementary file 3 [file Data_Sheet_3.PDF]

A

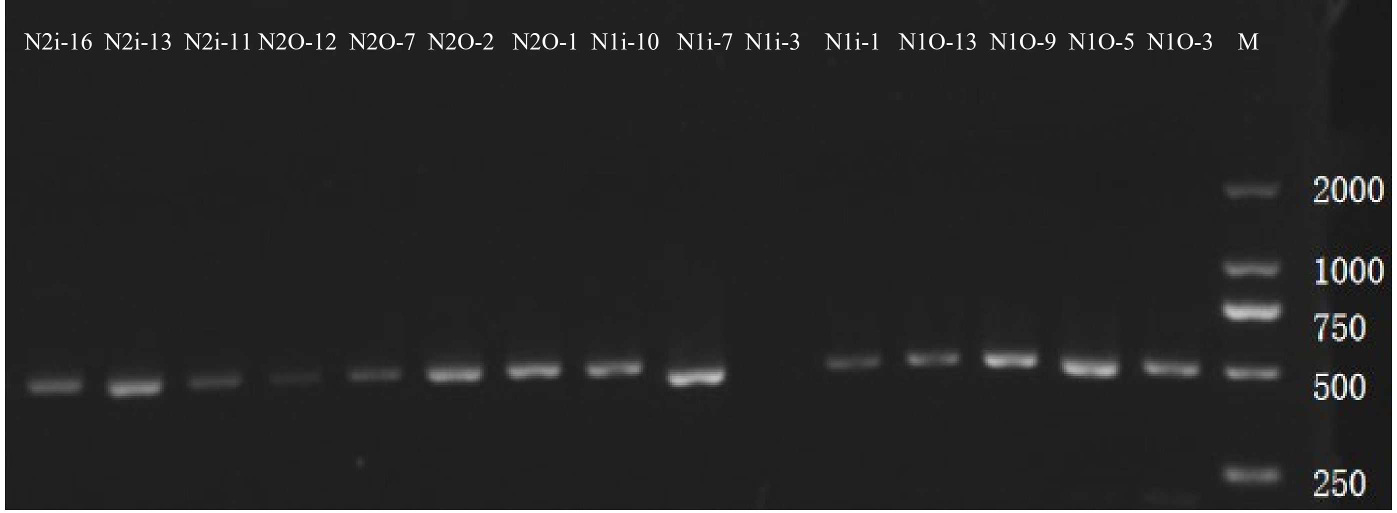

B

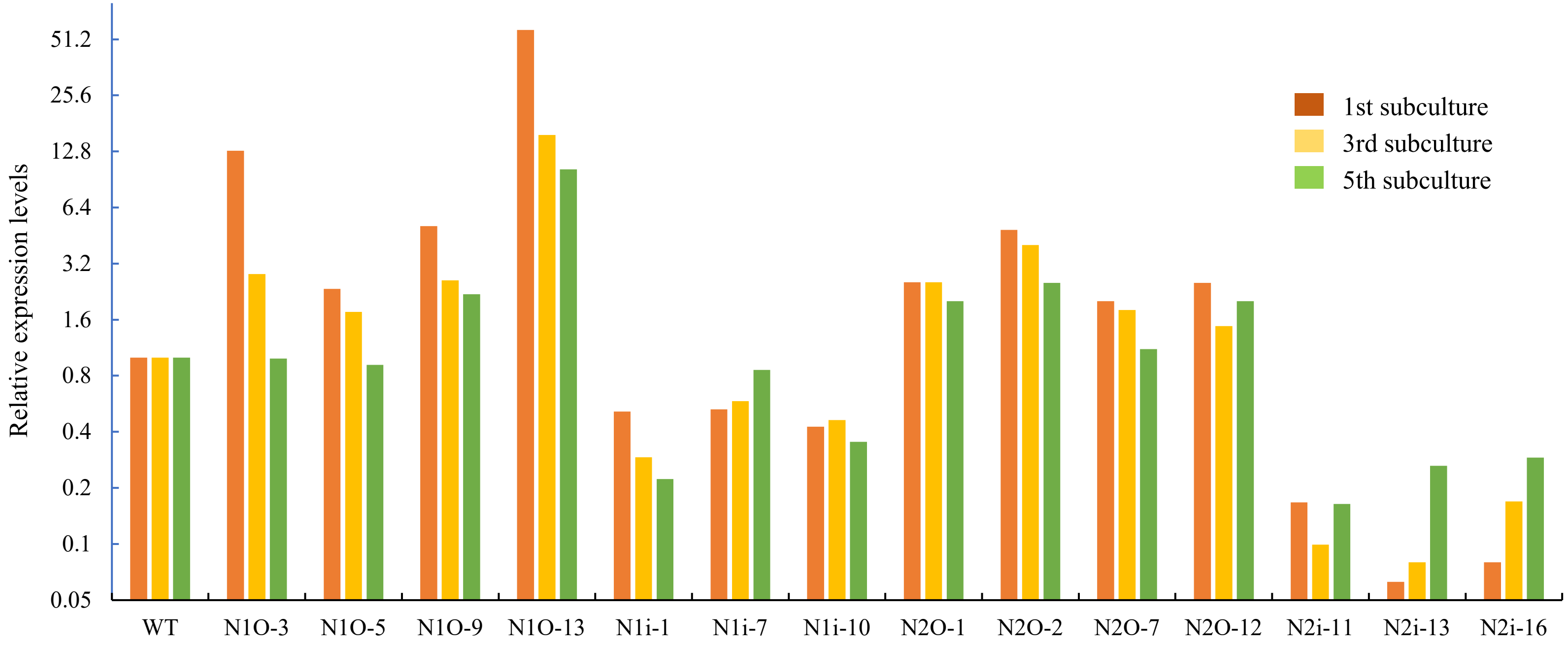

Figure S1 Identification of the foreign DNA fragments in randomly chosen transformants after having subcultured five times on PDA plates for the hereditary stability. (A) Detection of the *HygB* gene was conducted using 2% agarose gel electrophoresis; (B) Expression analysis of Noxs transformants by qRT-PCR.

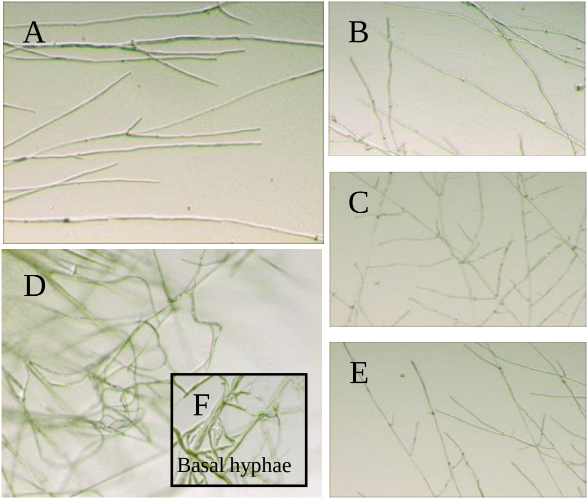

Figure S2 The morphological changes in mycelial formation and branches of *P. ostreatus* Nox mutants. (A-E) Photographs of the WT, PoNoxA^{OE}, PoNoxARNAi, PoNoxB^{OE} and PoNoxBRNAi strains growing on the CYM medium, respectively. (F) Photograph of basal hyphae of the PoNoxB^{OE} strain.

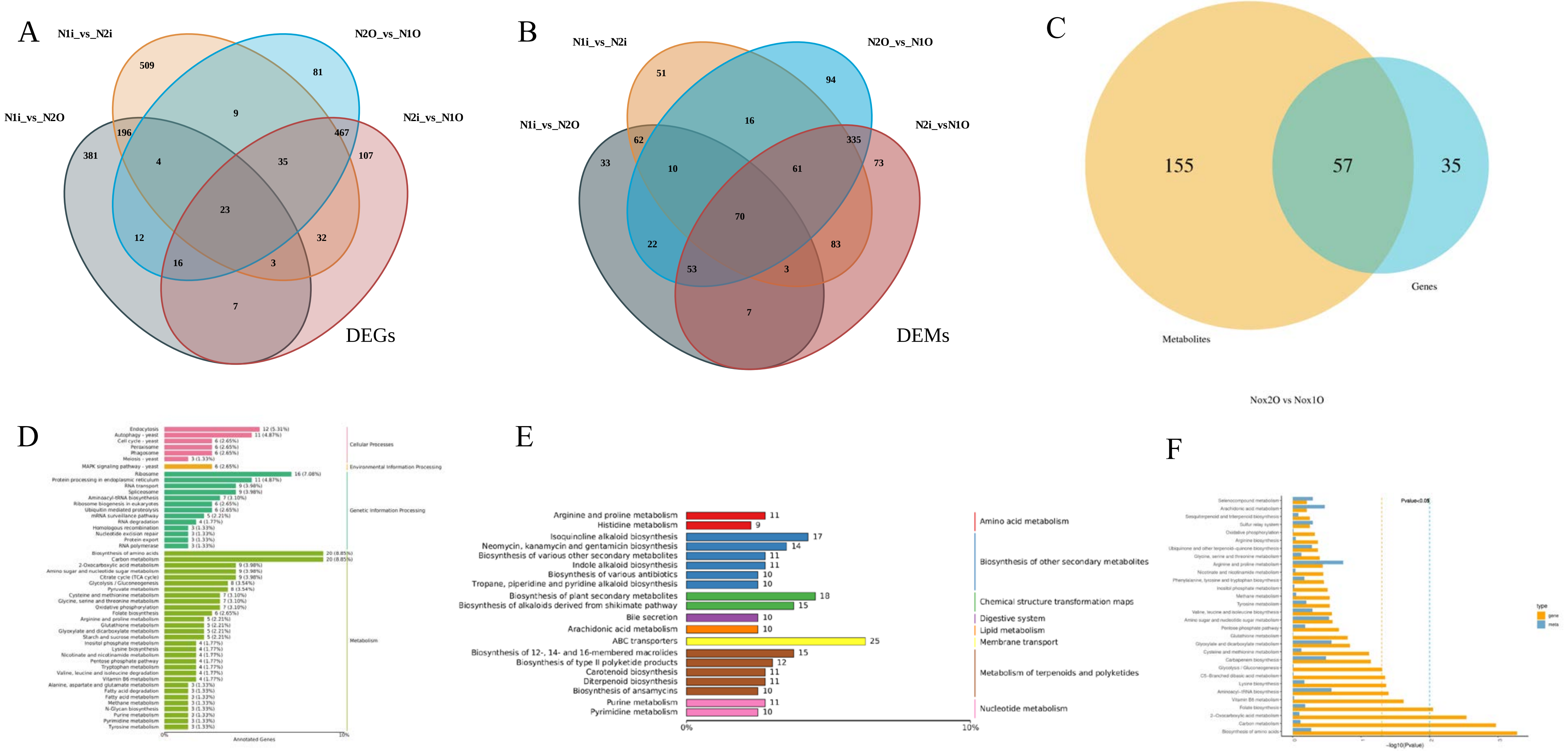

Figure S3 Expression analysis of genes, metabolites between the *PoNoxA* and *PoNoxB* overexpression and knockdown. (A-B) The Venn diagram shows the overlap between sets of differentially expressed genes (DEGs) and metabolites (DEMs) between the *PoNoxA* and *PoNoxB* mutants, respectively. (C)The Venn diagram shows the co-expressed DEGs and DEMs in the transcriptomics and metabolomics after the overexpression of *PoNoxA* and *PoNoxB* by KEGG analysis (D-E) Summarized main KEGG pathways of DEGs and DEMs between *PoNoxA* overexpression (N1O) and *PoNoxB* overexpression (N2O), respectively. (F) Combined metabolome and transcriptome analysis of *PoNoxA* overexpression (N1O) and *PoNoxB* overexpression (N2O) by KEGG enrichment analysis.
