# Supplementary figures and images for: Contrasting effects of NADPH oxidases on the fungal hyphae growth and immune responses in Pleurotus ostreatus
Source: Front Microbiol. 2024 Jun 19;15:1387643. doi: 10.3389/fmicb.2024.1387643 (PMC11220167; doi:10.3389/fmicb.2024.1387643)

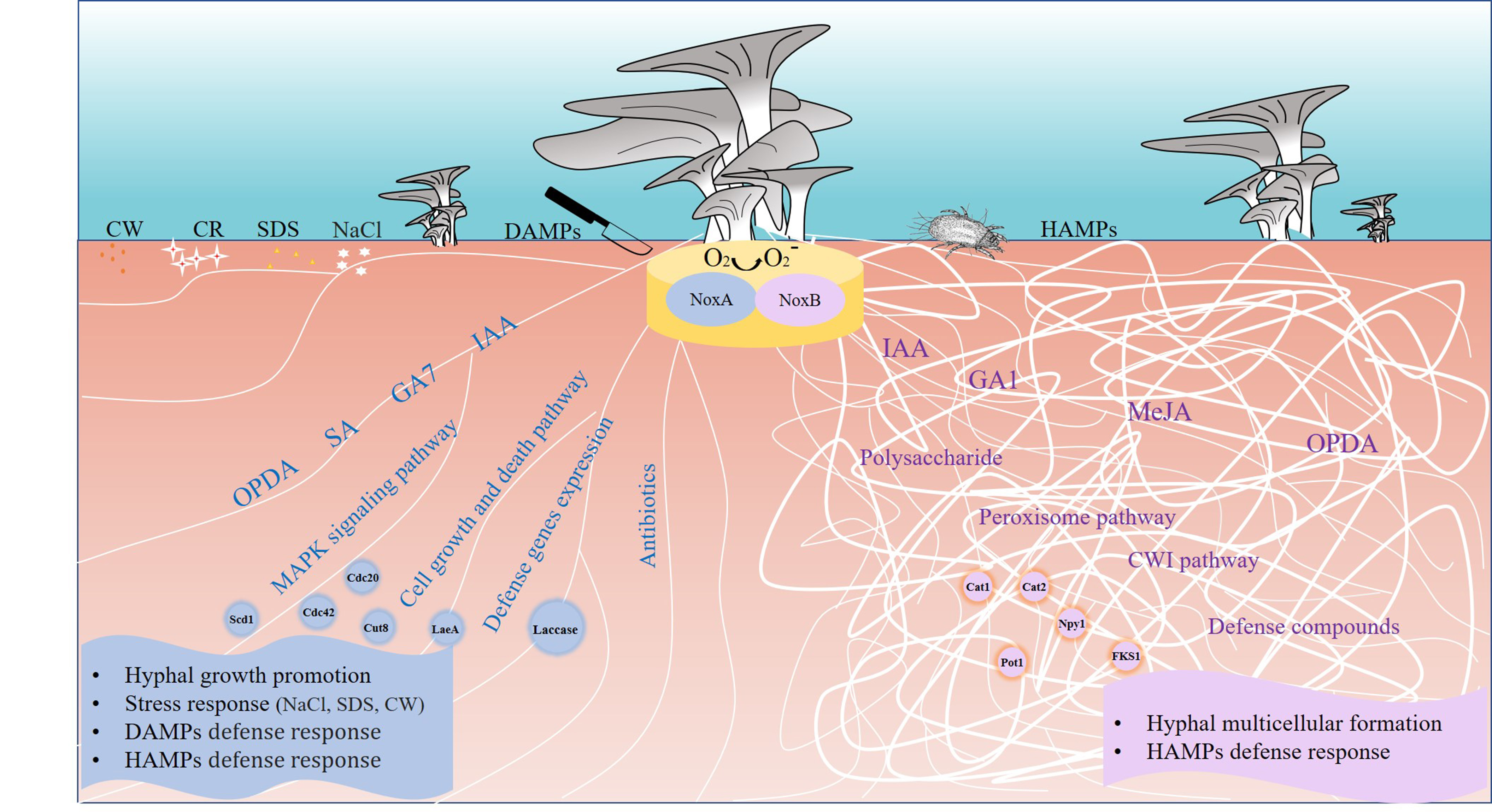

Supplement: Supplementary file 5 [file Image_1.JPEG]
